# TCCDNet: A Multimodal Pedestrian Detection Network Integrating Cross-Modal Complementarity with Deep Feature Fusion

**DOI:** 10.3390/s25092727

**Published:** 2025-04-25

**Authors:** Shipeng Han, Chaowen Chai, Min Hu, Yanni Wang, Teng Jiao, Jianqi Wang, Hao Lv

**Affiliations:** 1 School of Biomedical Engineering, Air Force Medical University, Xi’an 710032, China; sp.han2022@outlook.com (S.H.); ccw_0824@xauat.edu.cn (C.C.); hummmin@163.com (M.H.); jiaoteng@fmmu.edu.cn (T.J.); wangjq@fmmu.edu.cn (J.W.); 2 School of Information and Control Engineering, Xi’an University of Architecture and Technology, Xi’an 710055, China

**Keywords:** pedestrian detection, multimodal fusion, cross-modal information complementarity, feature fusion

## Abstract

Multimodal pedestrian detection has garnered significant attention due to its potential applications in complex scenarios. The complementarity characteristics between infrared and visible modalities can enhance detection performance. However, the design of cross-modal fusion mechanisms and the in-depth exploration of inter-modal complementarity still pose challenges. To address this, we propose TCCDNet, a novel network integrating cross-modal complementarity. Specifically, the efficient multi-scale attention C2f (EMAC) is designed for the backbone, which combines the C2f structure with an efficient multi-scale attention mechanism to achieve feature weighting and fusion, thereby enhancing the model’s feature extraction capacity. Subsequently, the cross-modal complementarity (CMC) module is proposed, which enhances feature discriminability and object localization accuracy through a synergistic mechanism combining channel attention and spatial attention. Additionally, a deep semantic fusion module (DSFM) based on a cross-attention mechanism is incorporated to achieve deep semantic feature fusion. The experimental results demonstrate that TCCDNet achieves a MR^−2^ of 7.87% on the KAIST dataset, representing a 3.83% reduction compared to YOLOv8. For the other two multimodal pedestrian detection datasets, TCCDNet attains mAP_50_ scores of 83.8% for FLIR ADAS and 97.3% for LLVIP, outperforming the baseline by 3.6% and 1.9% respectively. These results fully validate the effectiveness and advancement of the proposed method.

## 1. Introduction

Pedestrian detection, a key task in computer vision [1], plays an important role in applications such as autonomous driving [2], intelligent surveillance [3], and smart transportation. Accurate pedestrian detection technology can significantly enhance traffic safety and operational efficiency, for example, by preventing traffic accidents to ensure the safety of road users or optimizing signal control through real-time pedestrian flow monitoring to improve traffic efficiency. In the development of pedestrian detection technology, research on the application of RGB and infrared (IR) images has received significant attention. RGB images provide rich color, texture, and shape information under favorable lighting conditions, while IR images can clearly capture object contours in low-light or dark environments and exhibit strong penetration capabilities through obstacles like smoke. However, single-modal approaches have limitations. RGB image quality degrades significantly in low-light conditions, while IR images lack detailed color and texture information. Therefore, combining the advantages of both modalities to achieve feature complementarity has become a key direction for improving pedestrian detection performance.

In multimodal pedestrian detection tasks, selecting an appropriate network architecture and multimodal fusion strategy is crucial for improving the model’s detection performance. Compared to single-stream models, two-stream models can handle multispectral data more effectively while preserving the unique characteristics of each modality, making them the preferred architecture. This architecture not only offers greater flexibility and robustness but also adapts better to complex detection scenarios. For instance, studies by Guan et al. [4] and Chen et al. [5] have demonstrated the superior performance of two-stream network structures under varying lighting conditions. Regarding fusion strategies, feature-level fusion is one of the most commonly used multimodal fusion methods because it can better capture the complementarity between modalities, as highlighted by many researchers [6]. This method first extracts meaningful features (such as edges, colors, and textures) from the raw data and then integrates these features by combining complementary information from different sources to enhance target recognition accuracy. Specifically, this approach allows the model to leverage the color and texture information provided by RGB images and the thermal distribution information from IR images, thereby improving detection capabilities in complex environments [7].

Despite significant progress in multimodal pedestrian detection, challenges remain in preserving detailed features, aligning cross-modal features, and achieving robust modal interaction. To address these issues, this study proposes an innovative multimodal pedestrian detection framework named TCCDNet. The framework adopts a systematic design, optimizing three key aspects: feature extraction, cross-modal complementarity, and deep fusion. Experimental results on multiple public datasets demonstrate that our method offers significant advantages over previous approaches. The main contributions of this work are as follows:

- A novel TCCDNet architecture is proposed. The network is designed based on YOLOv8 with a dual-branch backbone to efficiently process visible and infrared multimodal data in a parallel architecture. It innovatively introduces an EMAC, which employs an adaptive feature weighting mechanism, enhancing the discriminative ability of modal features. This not only improves the capture of detailed information from RGB and infrared images but also effectively addresses the degradation of RGB image quality under low-light conditions, providing richer feature representations.
- The CMC module integrates channel attention and spatial attention mechanisms to focus on extracting and fusing key features from dual-modal data. This process not only enhances the discriminative power of feature representation but also achieves inter-modal feature complementarity at different scales. In this way, the CMC module maximizes the complementary information between the two modalities while preserving the unique strengths of each modality.
- The DSFM module achieves the deep integration of RGB and IR image features through a multi-level feature interaction mechanism. It adopts a progressive fusion strategy, optimizing the feature fusion process across different scales via an adaptive weight allocation mechanism. This significantly improves the accuracy and robustness of pedestrian detection. Compared to traditional methods that perform fusion only at the pixel or decision level, DSFM establishes finer inter-modal feature correlations, delivering more precise detection results.
- The proposed approach was rigorously evaluated on three benchmark multispectral datasets: KAIST, FLIR ADAS, and LLVIP. The experimental results indicate that the framework achieves an optimal trade-off between computational efficiency and detection accuracy, highlighting its potential for real-world applications.

## 2. Related Works

### 2.1. Unimodal Pedestrian Detection

Unimodal pedestrian detection techniques are mainly divided into two categories according to sensor type: RGB-based pedestrian detection and IR-based pedestrian detection. Early RGB-based methods mainly relied on handcrafted feature extraction techniques, particularly the combination of a histogram of oriented gradients (HOG) and linear support vector machines (SVMs), which dominated the field for a considerable period. For instance, Wang et al. [8] proposed an enhanced HOG feature descriptor combined with boosting algorithms to distinguish pedestrians from background objects. However, these traditional methods exhibit limited performance in complex environments, struggling to meet the demands of diverse real-world applications. With the advent of deep learning, convolutional neural network (CNN)-based techniques have gradually taken the lead due to their superior feature representation and learning capabilities, replacing conventional approaches. Currently, pedestrian detection methods using single-shot multibox detector (SSD) [9,10], YOLO [11], and Faster R-CNN [12] have become widely adopted. YOLO is particularly praised for its speed and accuracy, making it one of the top-performing algorithms. In addition, with the development of the transformer architecture, it has also begun to show great potential in the field of pedestrian detection [13,14]. Transformer, with its unique self-attention mechanism, is able to better capture long-range dependencies, further improving the accuracy and robustness of pedestrian detection in various complex scenarios. Although these methods have made significant progress in computational efficiency and detection accuracy, they still face challenges related to specific lighting conditions—RGB-based detection performs well during the day but struggles at nighttime or in low-light environments.

In contrast, IR-based detection captures thermal radiation emitted by objects, making it immune to lighting conditions. It maintains stable performance in low-light or no-light environments, providing significant advantages at nighttime and in other challenging scenarios. In recent years, researchers have proposed various innovative IR-based pedestrian detection methods. For example, Teutsch et al. [15] developed a real-time IR detection approach, Chen et al. [16] designed an attention-guided encoder–decoder architecture, and Zhao et al. [17] achieved cross-domain sharing of RGB and IR information through improved UNet and YOLO. While these efforts partially address specific challenges, their application scope remains limited. Zhang et al. [18] proposed an optimized version of YOLOv8 for nighttime IR detection, combining deformable convolution and partial channel convolution to construct the C2f FastD module and introducing an SE channel attention layer to improve detection accuracy and speed. Hao et al. [19] adopted a cross-scale feature fusion mechanism combined with a hierarchical attention mapping framework, effectively addressing issues such as multi-scale variations, partial occlusion, and environmental interference. Additionally, Zhang et al. [20] developed a multi-task learning architecture integrating segmentation and domain adaptation, using UNet and Swin Transformer for semantic segmentation to establish spatial constraints for enhanced pedestrian detection. Wang et al. [21] proposed PPDet, achieving outstanding detection performance through a pixel-level prediction strategy.

Despite the fact that the aforementioned methods address certain specific problems, their application is still constrained by the lack of rich, detailed information provided by RGB images. This limitation highlights the importance of exploring multimodal fusion strategies. By combining RGB and IR data, the strengths of both modalities can be fully utilized, enabling stable performance across various lighting conditions while capturing detailed visual information to meet more diverse application needs.

### 2.2. Multimodal Pedestrian Detection

Pedestrian detection methods based on multimodal fusion improve detection accuracy and reliability by integrating data from different modalities, such as RGB and IR, and leveraging their complementary characteristics to overcome the limitations of single-modal approaches. In this process, selecting the appropriate fusion stage and adopting an effective fusion strategy are crucial for enhancing the performance of multimodal pedestrian detection. Early research primarily focused on exploring different fusion stages. For instance, Wagner et al. [22] compared early and late fusion strategies within the R-CNN framework and found that late fusion demonstrated superior performance. Subsequent studies, such as those on Halfway fusion [23] and fusion RPN [24], further showed that intermediate fusion is more effective than both early and late fusion in many cases. Specifically, early fusion [25] operates directly at the image level, which tends to lose critical information and leads to degraded detection performance. In contrast, intermediate fusion achieves fine-grained information sharing at the feature level, significantly improving detection accuracy. Late fusion, implemented at the decision level, also outperforms early fusion. Thus, preserving the detailed features of the source images is essential for the success of multimodal fusion.

To achieve more efficient multimodal pedestrian detection, researchers have focused on optimizing feature fusion methods between different modalities and designing various high-performance cross-modal complementary modules. These efforts have focused on feature selection and alignment, fusion guided by attentional mechanisms, and cross-modal feature aggregation. First, in the aspect of feature selection and alignment, Zhou et al. [26] proposed a feature alignment module and a differential modality-aware fusion (DMAF) module, which can adaptively select the most suitable features based on lighting conditions. AR-CNN [27] enhances robustness to positional shifts by designing a regional feature alignment (RFA) module, addressing the impact of image registration issues on detection performance. Second, attention mechanism-guided fusion strategies have been widely applied. A. Althoupety et al. [28] developed a dual-attention feature fusion (DaFF) method that uses attention mechanisms to guide the entire fusion process. Lee et al. [29] introduced a novel cross-guided attention mechanism that leverages attention maps generated from inter-modal complementary interactions to enhance the representation power of multimodal features. Zhang et al. [30] proposed a cross-modal interactive attention network (CIAN), which also employs attention mechanisms to achieve the effective fusion of intermediate IR and RGB features. Finally, for cross-modal feature aggregation, Li et al. [31] constructed a multi-scale cross-modal homogeneity enhancement framework that performs multispectral feature fusion by measuring intra- and inter-modal interaction confidence. Chan et al. [32] designed a dual-stream model based on YOLO, enhancing complementarity between different spectra through adaptive multispectral weight adjustment. Junhwan Ryu et al. [33] proposed a multispectral interaction convolutional neural network (MICNN) that achieves inter-modal information exchange through feature map weight swapping. Yan et al. [34] designed a cross-modal complementary information fusion module that strengthens the interaction between the two modalities while retaining useful details from each. Additionally, some researchers have noted the significant impact of alignment issues in multispectral pedestrian image pairs and their labels on detection performance. MLPD [35] adopts a multi-label learning approach, assigning different labels to different input states to effectively handle incomplete pairing issues.

Multimodal fusion methods, by integrating the strengths of RGB and IR images, have significantly improved pedestrian detection capabilities. Therefore, this study systematically addresses and fuses RGB and IR image information by focusing on three key aspects: feature extraction, cross-modal information complementarity, and feature fusion. The goal is to maximize the utilization of multimodal data, thereby optimizing and enhancing pedestrian detection performance.

## 3. Methods

### 3.1. Structure of TCCDNet

**Figure 1:**
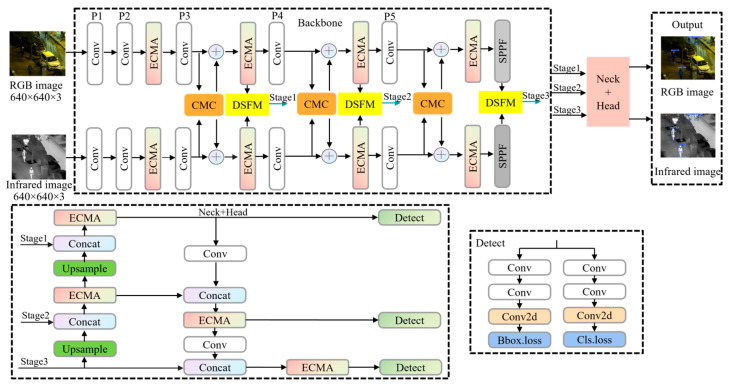
Overall architecture of TCCDNet. The diagram highlights the dual-branch backbone designed for efficient feature extraction, the neck component responsible for integrating features from both branches, and the head section dedicated to final output generation or decision making.

The architectural framework of the proposed TCCDNet is shown in Figure 1. The backbone of this network is extended based on YOLOv8, forming a dual-branch structure to construct the baseline model. The input stage processes pair RGB and IR images with dimensions of 640 × 640 × 3 through their respective branches, which follow symmetric processing pipelines. Each branch initially performs two convolution operations, each comprising a convolutional layer, batch normalization, and sigmoid activation. Feature extraction then proceeds through the EMAC module, followed by an additional convolution operation. At the P3 stage, cross-modal complementarity processing occurs in the CMC module, with the enhanced features being fused with the original branch features. Subsequently, EMAC further optimizes the features before they enter the DSFM module for deep semantic fusion. Finally, the fused features from the P3, P4, and P5 stages are processed through the neck network and detection head to generate the final detection results.

### 3.2. Efficient Multi-Scale Attention C2f Module

**Figure 2:**
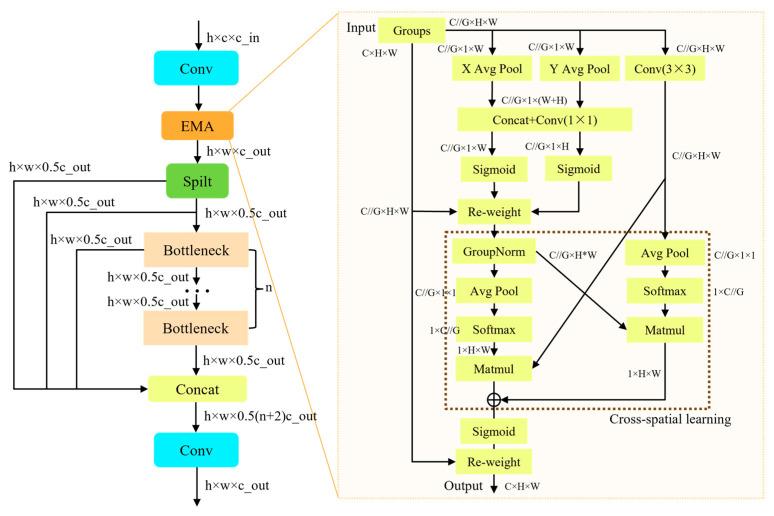
The architecture of the EMAC module, illustrating its key components and operational flow. On the right, the specific structure of the EMA module is depicted in detail.

The backbone of TCCDNet adopts an innovative hierarchical feature extraction architecture, which achieves efficient multi-level feature extraction and optimization from local details to global semantics through the alternating stacking of carefully designed convolutional layers and the EMAC module. This design enhances the model’s ability to understand complex patterns. The core innovation of the network lies in the design of the EMAC module, which optimizes the original excellent feature reuse characteristics of the C2f framework while innovatively integrating the EMA mechanism. This mechanism significantly enhances the model’s ability to capture multi-scale information and improves the modeling of long-range dependencies. The detailed architecture of EMAC is depicted in Figure 2.

The core module of EMAC, the EMA module, is illustrated on the right side of Figure 2. Unlike commonly used coordinate attention and spatial attention, the EMA module enhances feature representation through parallel sub-structures, addressing the limitations brought by traditional sequential processing methods and excessive network depth. It achieves effective channel description learning without reducing the channel dimension while also generating pixel-level attention for high-level features. Specifically, the module first splits the input features along the channel dimension into several sub-features, with each sub-feature responsible for learning different semantic information. Subsequently, the EMA module utilizes three parallel paths to generate attention weight descriptors for the grouped features. Two of the paths are located in the 1 × 1 convolution branch, focusing on capturing cross-channel dependencies, while the third path is in the 3 × 3 convolution branch, aiming to extract multi-scale feature representations. During the cross-spatial learning phase, EMA employs 2D global average pooling to process the output of the 1 × 1 branch, encoding global spatial information. Meanwhile, the output of the 3 × 3 branch is transformed into the corresponding dimensions and further processed through a unified activation mechanism. Finally, a spatial attention map that preserves precise spatial location information is generated using the softmax function. The output features within each group are aggregated by combining the two spatial attention weights generated from the two paths. These weights are computed using the sigmoid function, enabling the simultaneous capture of pixel-level pairwise relationships and global contextual information. The EMA module not only effectively models long-range dependencies but also embeds precise positional information, significantly enhancing the model’s feature representation capabilities. Its design is particularly well-suited for feature aggregation in complex scenarios, enabling the model to better handle various computer vision tasks.

### 3.3. Cross-Modal Complementarity Module

**Figure 3:**
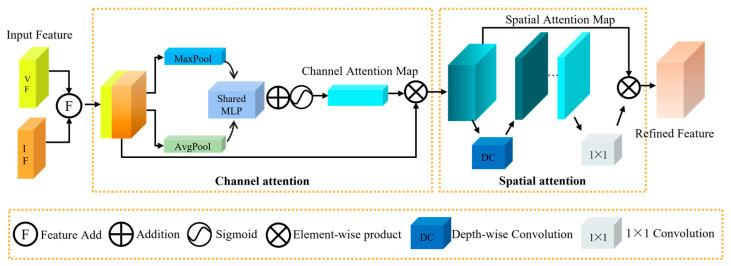
The CMC architecture illustrates the fusion of IR and RGB features via channel and spatial attention mechanisms.

In the field of pedestrian detection, effectively fusing the salient features of IR images with the texture details of RGB images to achieve cross-modal complementarity advantages remain a critical challenge. To address this issue and enhance both the accuracy of pedestrian detection and feature representation capabilities, this paper introduces an innovative cross-modal complement (CMC) module, the core architecture of which is depicted in Figure 3. The CMC module adopts a design that combines channel attention mechanisms with spatial attention mechanisms. By adaptively reweighting feature channels, the channel attention mechanism increases the model’s sensitivity to key information [36]. Meanwhile, the spatial attention mechanism emphasizes important regions in the image, improving the model’s target localization accuracy [37]. This combination not only preserves the integrity of the original features but also enhances the discriminability and robustness of the features through effective information integration, making it particularly suitable for pedestrian detection tasks in complex scenarios.

During CMC’s feature processing, the IR features IF and RGB features VF are initially fused through the feature summing operation to generate the fused features CF. This design, inspired by residual connections, preserves the integrity of the original features while enhancing their discriminative power through cross-modal information integration. The operation can be denoted as follows:

(1) CF=F(IF,VF)

The fused features CF are processed through the channel attention mechanism to generate the channel attention map. Complementarity features are first formed through the max pooling (MaxPool) and average pooling (AvgPool) layers. MaxPool enhances the model’s ability to identify critical features by retaining the maximum value within each window, thereby emphasizing significant features. However, this process can result in a partial loss of detail. To address this limitation, AvgPool is introduced to compute the average of feature values within the window, thereby preserving more global detail information. The dual-pooling strategy effectively mitigates the information loss associated with single pooling operations while capturing multi-granularity spatial features. The pooled features are further processed through a shared multilayer perceptron (MLP) for feature extraction and learning, and the outputs of the shared MLP are integrated via element-wise summation. The features are then nonlinearly transformed via the sigmoid function to generate channel attention features. These are multiplied element-wise with the original features, merging the original detail information with channel-enhanced salient features to produce the comprehensive, semantically rich features $\hat{F}$. Specifically, the original features retain the complete spatial structure and detailed information of the input signals, while the channel attention features strengthen the key feature channels and important regions. The element-wise multiplication enables feature complementarity, emphasizing key features while preserving detail integrity, thus providing more discriminative representations for subsequent detection tasks. The expression is as follows:

(2) $$\hat{F}=\mathrm{CF}⊗(\mathrm{Sigmoid}(\mathrm{MLP}(\mathrm{AvgPool}(\mathrm{CF}))+\mathrm{CL}(\mathrm{MaxPool}(\mathrm{CF}))))$$

To further enhance feature representation, the features $\hat{F}$ are fed into a spatial attention mechanism. This mechanism effectively captures the relative spatial positions of pedestrian targets in dense scenes. Compared to single-dimensional spatial weight allocation, dynamically assigning attention weights across both channel and spatial dimensions yields superior results. During depthwise convolution, each input channel independently applies a convolutional kernel to capture spatial relationships between features. This process maintains inter-channel correlations while simultaneously reducing computational complexity. The spatial attention module’s output undergoes channel mixing through a 1 × 1 convolution, generating a more refined attention map. Finally, the features processed by the channel attention mechanism are progressively multiplied with the unprocessed features, producing the final optimized features RF. The operation can be described by the following equation:

(3) $${\mathrm{RF}=\mathrm{Conv}}_{1\times 1}\left(\mathrm{DC}(\hat{F})\right)⊗\hat{F}$$

where ${\mathrm{Conv}}_{1\times 1}$ stands for the 1 × 1 convolution, DC represents the depthwise convolution, and ⊗ signifies the element-wise multiplication operation.

### 3.4. Deep Semantic Fusion Module

**Figure 4:**
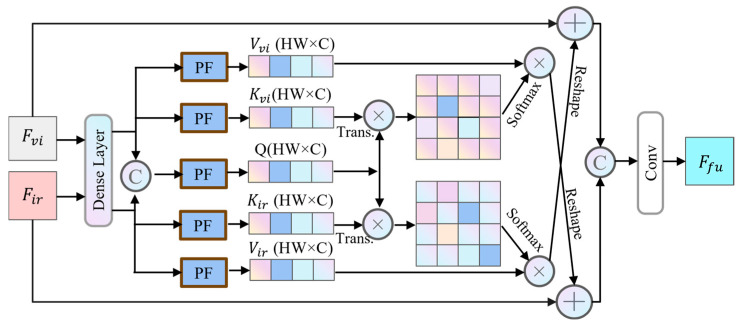
The architecture of the DSFM. This module is specifically designed to integrate deep semantic features from infrared and visible light images.

The deep semantic fusion module (DSFM) is designed to process the deep semantic features of RGB and IR images. This module first concatenates the features of these two modalities along the channel dimension, and then generates attention weights through convolution and pooling operations. This approach enables more effective utilization of the complementary characteristics present in multimodal information. The detailed structure of DSFM is shown in Figure 4.

The DSFM first enhances the IR and RGB features by integrating features extracted from the backbone through densely connected layers, forming high-level feature representations. It then outputs the enhanced IR feature $F_{ir}^{i}$ and RGB feature $F_{vi}^{i}$. Subsequently, a projection function PF, utilizing convolution and reshaping operations, transforms these features into key–value pairs $K_{m}^{i},V_{m}^{i}$. The operations are defined as follows:

(4) $$K_{m}^{i}=\mathrm{Reshape}({\mathrm{Conv}}_{K}^{m}(F_{m}^{i}))$$

(5) $$V_{m}^{i}{=\mathrm{Reshape}(\mathrm{Conv}}_{V}^{m}(F_{m}^{i}))$$

where $m\in \left\{ir,vi\right\}$ represents both IR and RGB modes, $K_{m}^{i}\in R^{C_{i}\times H_{i}\times W_{i}}$ denotes the key, and $V_{m}^{i}\in R^{C_{i}\times H_{i}\times W_{i}}$ denotes the value, with *C*, *H*, and *W* representing the number of channels, height, and width of the feature. Conv and Reshape refer to the convolution and reshaping operations, respectively. At this stage, the query vectors Q of the RGB and IR images are merged to perform a joint query operation, enabling the model to simultaneously consider query information from both modalities and more comprehensively capture and integrate their feature information. Specifically, the joint query allows the model to fully leverage the complementarity and interrelationships between the two modalities during feature extraction and information fusion. This ultimately enhances the model’s recognition and detection capabilities for objects. The operation can be denoted as follows:

(6) $$Q^{i}=\mathrm{Reshape}(\mathrm{Conv}(\mathrm{Concat}(F_{ir}^{i},F_{vi}^{i})))$$

where $Q^{i}\in R^{C_{i}\times H_{i}\times W_{i}}$ represents the query and Concat denotes the concatenation operation. Subsequently, the query vector *Q* is multiplied by the transposed matrix of the key vector *K* to compute the attention score map. This score map reflects the strength of relationships between different positions. Next, the obtained attention scores are normalized using the softmax function, converting them into a probability distribution. This probability distribution represents the attention weights of each position relative to others, indicating the relative importance of each position’s features during feature aggregation. Through this mechanism, the model dynamically allocates attention resources to different positions, enabling an adaptive focus on critical information within the feature. The operation is formulated as follows:

(7) $$P_{m}^{i}=\mathrm{Softmax}(Q^{i}{K_{m}^{i}}^{T})$$

where $P_{m}^{i}\in R^{H_{i}W_{i}\times H_{i}W_{i}}$ stands for the attention score map. Subsequently, the obtained attention probability distribution is multiplied element-wise with the value vector V, yielding attention scores for each object. This process assigns weights to feature vectors at each position based on their importance, emphasizing critical features while suppressing less relevant ones. After computing the attention scores, a reshape operation adjusts the feature maps to the appropriate dimensions, ensuring compatibility for additive fusion with the other modality’s features. This enables the effective integration and complementarity of the two modalities’ features. The attention output is then added to the input features via a residual connection, mitigating the vanishing gradient problem in deep networks. Finally, the bimodal feature maps are concatenated. A convolutional layer is applied to further process the features, extracting higher-level representations. Finally, the fused feature $F_{fu}^{i}$ is output. The operation is mathematically represented as follows:

(8) $$F_{fu}^{i}=\mathrm{Conv}(\mathrm{Concat}(F_{vi}^{i}⊕\mathrm{Reshape}(P_{ir}^{i}V_{ir}^{i},F_{ir}^{i}⊕\mathrm{Reshape}(P_{vi}^{i}V_{vi}^{i}))),$$

## 4. Experiments and Discussion

### 4.1. Experimental Settings

#### 4.1.1. Datasets

The proposed TCCDNet is evaluated on three widely recognized public datasets: KAIST, LLVIP, and FLIR ADAS. These datasets are chosen for their comprehensive coverage of various pedestrian detection scenarios, enabling a thorough assessment of the network’s performance.

KAIST: The KAIST dataset contains 95,328 RGB and IR image pairs with 103,128 densely annotated instances. It covers various traffic scenarios, including campus, street, and rural environments, during both daytime and nighttime, with images at a 640 × 480 resolution. The annotations categorize objects into three classes: “person” for distinguishable individuals, “people” for groups that are difficult to distinguish, and “cyclist” for individuals on bicycles. For training, the improved person annotations provided by AR-CNN [27] are utilized, comprising 8963 image pairs. For evaluation, the enhanced KAIST test set by Liu et al. [23] is employed, comprising 2252 images sampled from test videos at 30-frame intervals, including 1455 daytime and 797 images nighttime images.

LLVIP: The LLVIP dataset comprises 30,976 images, which equates to 15,488 pairs of strictly aligned RGB and IR images, providing a high-quality data foundation for multimodal learning. The dataset is exclusively labeled with the “person” category, focusing on pedestrian detection tasks. Most images are captured in extremely dark environments, accurately replicating nighttime or low-light conditions, making it particularly valuable for nighttime pedestrian detection research. The dataset features precise pedestrian annotations, ensuring its suitability for pedestrian detection and related tasks. It encompasses diverse scenes, including 24 low-light and 2 daytime environments, thoroughly addressing variations in lighting conditions. In this experiment, 12,025 image pairs are allocated for training and 3463 for testing, ensuring the model’s generalization capability across different scenarios.

FLIR ADAS: The FLIR ADAS dataset, a multispectral object detection dataset covering both daytime and nighttime scenes, was curated for this study by manually removing misaligned image pairs to ensure data quality. The cleaned dataset comprises 5142 multispectral image pairs, with 4129 pairs used for training and 1013 pairs for testing. While the FLIR ADAS dataset includes annotations for various object categories such as pedestrians, people, cars, and bicycles, this experiment specifically utilizes pedestrian annotations to concentrate on pedestrian detection research.

#### 4.1.2. Implementation Details

The training environment for this experimental model consists of an Ubuntu 20.04 operating system with a TITAN X GPU. The deep learning framework used includes Pytorch 1.10 and CUDA 10.1. TCCDNet is trained using the SGD optimizer with an initial learning rate of 0.02 for 120 epochs and a default batch size of 4. During training, samples with an IoU greater than 0.5 are considered positive, while others are labeled as negative. To further enhance the model’s performance and generalization ability, several strategies are adopted. First, for learning rate scheduling, cosine annealing strategy is employed to dynamically adjust the learning rate, which helps the model escape local optima and accelerates convergence. Second, to increase the diversity of the training dataset and improve the model’s robustness, various data augmentation techniques are applied, including random cropping, horizontal flipping, color jittering, and mosaic augmentation. Additionally, to prevent gradient explosion and stabilize the training process, gradient clipping is implemented. A weight decay coefficient of 0.0005 is set to reduce overfitting and enhance the model’s generalization ability. The momentum factor is set to 0.937 to accelerate convergence and improve training stability.

#### 4.1.3. Evaluation Metrics

For the KAIST dataset, performance is evaluated using the log-average miss rate (L-AMR), calculated by averaging miss rates at false positives per image (FPPI) points sampled between 10^−2^ and 10^0^, denoted as MR^−2^. A lower MR^−2^ reflects superior model performance. For the FLIR ADAS and LLVIP datasets, the evaluation employs the widely used MS-COCO object detection metric, mean average precision (mAP), which offers a stable and comprehensive performance measure. Specifically, mAP_50_ and mAP_75_ represent mAP values at IoU thresholds of 0.5 and 0.75, respectively, while mAP is the average mAP calculated at IoU thresholds from 0.5 to 0.95 with a 0.05 interval. Higher mAP values indicate superior performance.

### 4.2. Qualitative Results and Analysis

#### 4.2.1. Results for the KAIST Dataset

This experiment validates the performance of the model on the reasonable subset of the KAIST [38] dataset. This subset filters pedestrian annotation samples to exclude heavily occluded instances and retain those with a height of at least 50 pixels. The filtered samples are further divided into three evaluation scenarios based on the time of capture, including reasonable-all, reasonable-day, and reasonable-night. Table 1 compares TCCDNet with nine mainstream multimodal pedestrian detection methods, including ACF [38], Halfway Fusion [23], Fusion RPN+BF [24], IATDNN+IASS [39], IAF-RCNN [40], CIAN [30], MSDS-RCNN [41], AR-CNN [27], and MBNet [26].

The results show that in the most challenging all-day and nighttime scenes, TCCDNet significantly outperforms other comparative methods, achieving L-AMRs of 7.87% and 6.43%, respectively, and ranking first in detection performance. In the daytime scenario, TCCDNet ranks second with an L-AMR of 8.66%, closely following MBNet’s 8.28%. Additionally, the model demonstrates excellent real-time performance, with a single-frame processing speed of 0.05 s. These findings fully highlight the advancements and superiority of the TCCDNet algorithm in multimodal pedestrian detection tasks, particularly under complex environmental conditions. The algorithm not only enhances detection accuracy but also demonstrates significant advantages in processing speed, providing robust and reliable support for practical applications.

**Figure 5:**
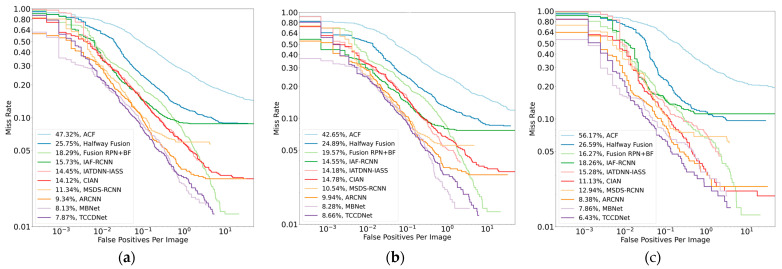
MR-FPPI plots of TCCDNet and other methods for KAIST under different settings. (**a**) Reasonable-all; (**b**) reasonable-day; (**c**) reasonable-night.

The MR-FPPI curves (IoU = 0.5) for the proposed TCCDNet, compared to those for other state-of-the-art methods, are illustrated in Figure 5 under the reasonable-all, reasonable-day, and reasonable-night settings on the KAIST dataset. The experimental results demonstrate that TCCDNet achieves remarkable performance across all settings, with L-AMRs of 7.87%, 8.66%, and 6.43% for the reasonable-all, reasonable-day, and reasonable-night subsets, respectively. Compared to the state-of-the-art MBNet model, which achieves L-AMRs of 8.13%, 8.28%, and 7.86% for the corresponding subsets, TCCDNet ranks first in both the reasonable-all and reasonable-night evaluations. Notably, TCCDNet exhibits significant improvements in nighttime detection, reducing the miss rate by 1.43% compared to that of MBNet while also achieving a 0.26% reduction in the reasonable-all setting. These results highlight that TCCDNet’s cross-modal feature fusion mechanism enables more robust learning of pedestrian characteristics, particularly under challenging nighttime conditions.

**Figure 6:**
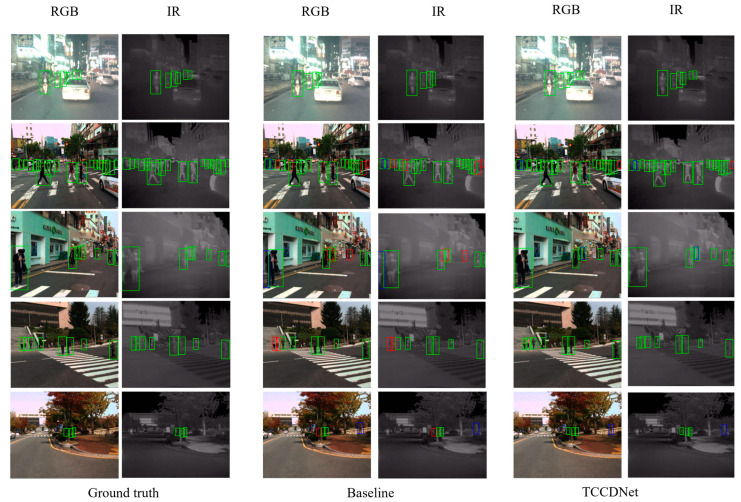
Visualization result comparison for the KAIST dataset. Green boxes denote correct detections, blue boxes mark false positives, and red boxes highlight missed detections.

To visually compare the detection performance of TCCDNet with the baseline that solely employs YOLOv8 to construct a dual-branch architecture, a python script was developed to analyze and output correct detections, missed detections, and false detections for each test image. This quantitative analysis method allows for the precise evaluation of each model’s performance in pedestrian detection tasks, providing valuable data to support subsequent model optimization. Figure 6 visualizes the pedestrian detection results for the KAIST dataset, with blue boxes indicating false detections and red boxes highlighting missed detections. The image in the second row depicts a dense scene with pedestrians at a traffic light. Compared to the baseline model, TCCDNet demonstrates superior performance in handling pedestrian detection in dense scenarios, with only one missed detection at the edge, whereas the baseline model has four missed detections. The images in the third and fourth rows show that TCCDNet achieves higher accuracy when detecting pedestrians standing side by side or slightly staggered. The results in the fifth row further demonstrate that TCCDNet outperforms the baseline in detecting pedestrians occluded by tree objects. These findings indicate that the added modules enable better learning of the characteristics of dense crowds, resulting in improved detection performance.

#### 4.2.2. Results for the FLIR ADAS Dataset

Table 2 compares TCCDNet with other methods using FLIR ADAS, including YOLOv5’s [42] single-modal RGB or IR and dual-modal approaches, GAFF [43] with VGG16 and ResNet18 backbones, and CFT [44]. YOLOv5’s single-modal results show that the IR modality outperforms the RGB modality, with mAP_50_ scores of 73.9% and 67.8%, respectively. However, the two-stream YOLOv5 achieves a lower mAP_50_ than the IR-only model at 73.0%, indicating that simple fusion strategies fail to fully leverage multimodal data advantages. The GAFF models with VGG16 and ResNet18 backbones show similar performance, with mAP_50_ scores of 72.7% and 72.9%, while the CFT model, with its custom CFB backbone, achieves a significant improvement with an mAP_50_ of 78.7%, highlighting the importance of tailored designs. TCCDNet outperforms all with mAP_50_, mAP_75_, and mAP scores of 83.3%, 36.2%, and 42.1%, surpassing CFT by 4.6%, 0.7%, and 1.9%, respectively. Its innovative design, including the EMAC module for enhanced feature extraction, the CMC module for cross-modal complementarity, and the DSFM module for deep semantic fusion, enables exceptional performance in complex environments. TCCDNet’s hierarchical attention mechanisms and multi-scale feature integration set a new benchmark for pedestrian detection, particularly in low-light or occluded scenarios, demonstrating strong practical utility.

**Figure 7:**
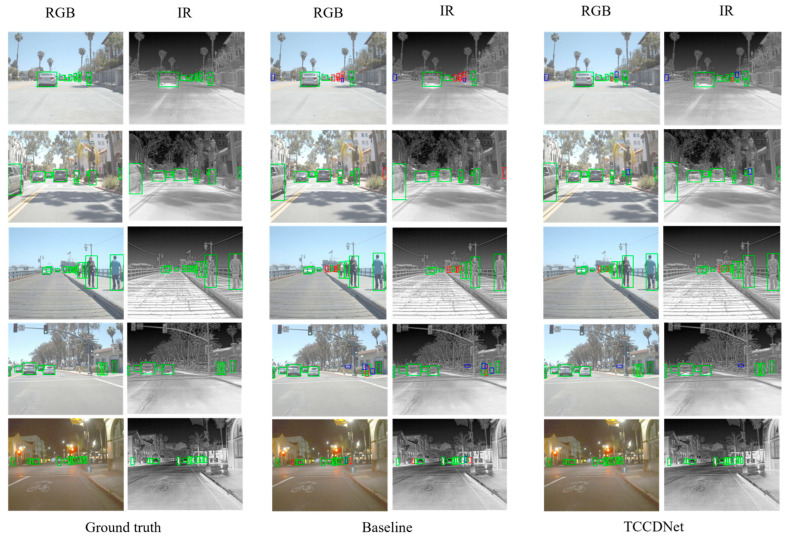
Visualization result comparison for FLIR ADAS. Green boxes represent correct detections, blue boxes mark false positives, and red boxes highlight missed detections.

To intuitively compare detection performance differences between TCCDNet and the baseline, Figure 7 presents the visualization results of detection for the FLIR ADAS test set. The first, second, and fifth rows demonstrate that TCCDNet outperforms the baseline in detecting edge objects and distant small objects, particularly excelling in object recognition against complex backgrounds. The third row depicts a scene with alternating near and far pedestrians, where TCCDNet, leveraging its strong spatial perception capabilities, demonstrates superior detection performance in cases of mutual occlusion among pedestrians, effectively reducing missed and false detections. The results in the fourth row further indicate that TCCDNet achieves more accurate detection in pedestrian occlusion scenarios by learning rich spatial semantic information through deep learning, showcasing its robustness and generalization ability in complex scenes. These visualization results not only validate the superior performance of TCCDNet but also provide strong support for its potential in practical applications.

#### 4.2.3. Results for the LLVIP Dataset

Table 3 presents the performance comparison results of TCCDNet for the LLVIP dataset. In this study, we selected YOLOv5’s [42] single-modal RGB and IR methods, the GAFF [43] method based on VGG16, CCIFNet [34], and CFT [44], among other representative methods, for comparative analysis. Due to the relatively low scene complexity of the LLVIP dataset, most models achieve mAP_50_ scores exceeding 90%, with some advanced models even surpassing 97% on this metric, leading to a gradual reduction in differentiation among models. Therefore, stricter evaluation metrics, namely mAP_75_ and mAP, are more reflective of a model’s precise detection capabilities. The experimental results show that TCCDNet performs exceptionally well across all three core metrics: the mAP_50_ reaches 97.3%, the mAP_75_ is 78.4%, and the mAP is 67.8%. Particularly for the more challenging mAP_75_ and mAP metrics, TCCDNet leads the second-place CFT by 5.5% and 4.2%, respectively, significantly outperforming other comparative methods. This is attributed to its enhanced spatial perception capabilities, which enable the precise localization of target boundaries and the capture of semantic features of occluded targets. Notably, although TCCDNet’s mAP_50_ is 0.3% lower than that of CCIFNet, its collaborative learning mechanism for local details and global semantics demonstrates unique advantages in high IoU threshold scenarios: by integrating multi-scale convolutional features, TCCDNet exhibits stronger robustness in small target detection and occluded pedestrian recognition tasks, thereby achieving significant improvements in mAP_75_ and overall mAP metrics. These results validate that TCCDNet maintains exceptional detection accuracy in complex scenarios, particularly excelling in the refinement of target localization.

**Figure 8:**
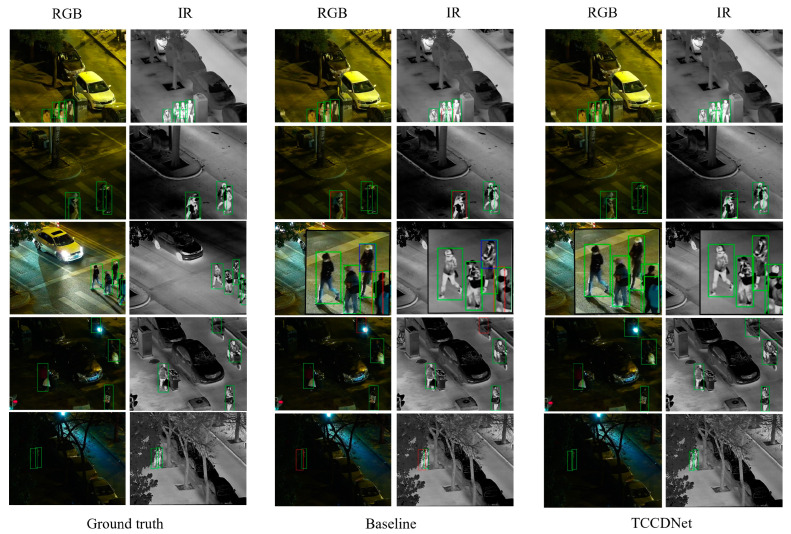
Visualization result comparison for LLVIP. Green boxes represent correct detections, blue boxes represent false positives, and red boxes indicate missed detections.

Figure 8 visually compares TCCDNet’s detection results for the LLVIP test set against ground truth and the baseline. From the results in the fifth row, it can be seen that TCCDNet is still able to achieve accurate recognition when pedestrians are partially occluded by objects such as trees. This indicates that the model excels in learning spatial information and local pedestrian features, effectively capturing key pedestrian characteristics under partial occlusion conditions. Additionally, from the results in the first, second, and third rows, it can be observed that even in scenarios with dense crowds, mutual occlusion, or incomplete body parts, TCCDNet maintains a high recognition accuracy. These results further validate the robustness and superiority of TCCDNet under challenging conditions such as complex scenes, dense pedestrian environments, and mutual occlusion.

### 4.3. Ablation Study

To validate the effectiveness of each module in the proposed algorithm, ablation experiments were conducted on the LLVIP, FLIR ADAS, and KAIST datasets, with detailed analysis of the results. Based on the results in Table 4, using a dual-branch CSPDarknet53 network as the baseline, we systematically evaluated the contribution of each component by incrementally integrating the CMC, DSFM, and EMAC modules. Specifically, when all three modules—CMC, DSFM, and EMAC—were integrated into the baseline model to form TCCDNet, the results indicated that each module significantly reduced the miss rate on the KAIST dataset. Compared to the baseline model, TCCDNet achieved a reduction in the miss rate of 3.83% under all-day conditions, 4.48% during the daytime, and 2.75% at night. Notably, the DSFM module contributed more to reducing the miss rate in nighttime conditions, while the EMAC module performed better during daytime. Through the synergistic effects of these modules, TCCDNet achieved superior detection performance in pedestrian detection tasks.

**Figure 9:**
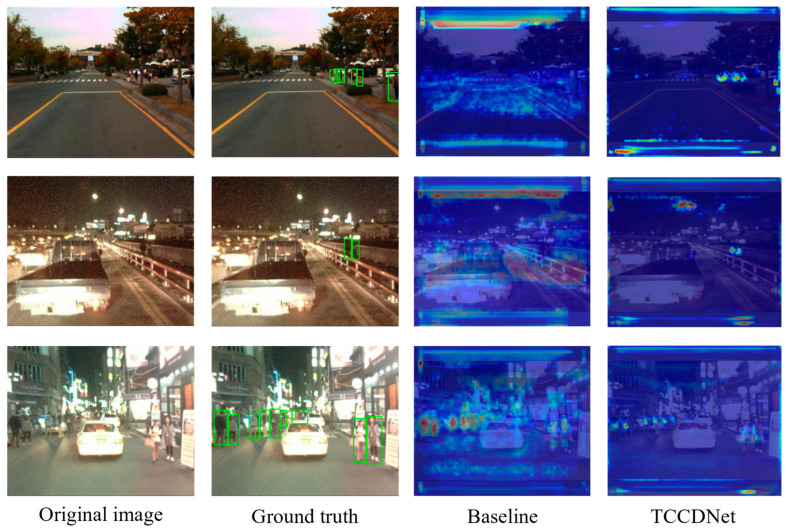
Visualization comparison of the attention mechanism of our TCCDNet with the baseline on the KAIST dataset using Grad-CAM.

To more intuitively compare the improvement effects of the models, we used the Grad-CAM method to visualize and compare the gradient information of the baseline model based on YOLOv8 and the improved TCCDNet for the KAIST dataset. As shown in Figure 9, the first column displays the original images, the second column shows the detection results with bounding boxes, the third column presents the attention maps generated by the baseline model, and the fourth column displays the attention maps produced by the improved TCCDNet. The results indicate that, compared to the baseline model, the improved model is able to more precisely highlight the critical regions in the images where pedestrians are located.

Table 5 reveals the optimization effects of TCCDNet on the FLIR ADAS dataset through ablation experiments. The results show that the integration of each module positively contributed to the network’s performance. Specifically, TCCDNet achieved a 3.6% improvement in mAP_50_, a 4.9% increase in mAP_75_, and a 3.2% boost in overall mAP compared to the baseline model. Meanwhile, the model’s precision and recall rates improved by 1.5% and 4.8%, respectively. These data fully validate the effectiveness of each module in TCCDNet and collectively highlight the model’s outstanding performance in pedestrian detection tasks.

Table 6 presents the ablation experiment results for the LLVIP dataset, highlighting the contributions of the CMC, DSFM, and EMAC modules to the baseline model. The CMC module provides the most significant improvements, increasing precision by 1.1%, recall by 3.4%, and mAP by 4.4%, showcasing its effectiveness in enhancing feature representation. The DSFM and EMAC modules also contribute notably, with EMAC achieving the highest precision gain at 1.3% and DSFM improving recall by 3.0%. When integrated into TCCDNet, these modules work synergistically to achieve the best performance, with recall reaching 93.1%, a 3.8% increase, and mAP_50_ improving to 97.3%, a 1.9% increase. These results demonstrate that each module improves the performance of diverse aspects of the detection task, with CMC and EMAC excelling in precision and DSFM boosting recall, collectively leading to superior overall detection performance.

## 5. Conclusions

This paper proposes TCCDNet, an innovative dual-branch network for RGB-IR multimodal pedestrian detection that systematically exploits cross-modal complementarity through three novel components. Specifically, the EMAC module enhances target representation via global–local collaborative perception, the CMC module establishes cross-modal calibration to maximize complementary information, and the DSFM module refines feature discriminability through hierarchical semantic fusion. Extensive experiments demonstrate state-of-the-art performance. TCCDNet achieves a miss rate of 7.87% on the KAIST dataset, outperforming YOLOv8 by 3.83%. On the FLIR ADAS and LLVIP datasets, it reaches mAP_50_ scores of 83.8% and 97.3% respectively, showing improvements of 3.6% and 1.9% over baseline models. These results validate TCCDNet’s robustness in complex scenarios while providing a computationally efficient solution for multimodal detection tasks. The proposed framework opens new possibilities for safety-critical applications such as autonomous driving and surveillance.

Despite the significant advancements achieved, our research does have some limitations. Currently, TCCDNet is primarily designed for specific sensor types, namely RGB and infrared, with limited support for integrating other data types such as LiDAR or radar. Future work can focus on expanding the framework to incorporate additional sensor data, thereby enhancing detection accuracy and reliability even further. Looking ahead, we plan to continue refining TCCDNet. Our efforts will not only address these current limitations but also explore new application domains. This will help to drive the development of more advanced multimodal perception technologies.

## Figures and Tables

**Table 1:** Comparison results for the KAIST dataset.

Model	Backbone	MR^−2^ (IoU = 0.5) (%)			Platform	Speed (s)
		All	Day	Night		
ACF [38]	—	47.32	42.65	56.17	MATLAB	2.73
Halfway Fusion [23]	VGG-16	25.75	24.89	26.59	TITAN X	0.43
Fusion RPN+ BF [24]	VGG-16	18.29	19.57	16.27	MATLAB	0.80
IATDNN + IASS [39]	VGG-16	14.95	14.67	15.72	TITAN X	0.25
IAF-RCNN [40]	VGG-16	15.73	14.55	18.26	TITAN X	0.21
CIAN [30]	VGG-16	14.12	14.78	11.13	1080Ti	0.07
MSDS-RCNN [41]	VGG-16	11.34	10.54	12.94	TITAN X	0.22
AR-CNN [27]	VGG-16	9.39	9.94	8.38	1080Ti	0.12
MBNet [26]	ResNet-50	8.13	**8.28**	7.86	1080Ti	0.07
TCCDNet (ours)	CSPDarknet53	**7.87**	8.66	**6.43**	TITAN X	**0.05**

Bold represents the best results.

**Table 2:** Comparison results for the FLIR ADAS dataset.

Model	Modality	Backbone	mAP_50_	mAP_75_	mAP
YOLOv5 [42]	RGB	CSPDarknet53	67.8	25.9	31.8
YOLOv5 [42]	IR	CSPDarknet53	73.9	35.7	39.5
Two-stream YOLOv5 [42]	RGB + IR	CSPDarknet53	73.0	32.0	37.4
GAFF [43]	RGB + IR	VGG16	72.7	30.9	37.3
GAFF [43]	RGB + IR	ResNet18	72.9	32.9	37.5
CFT [44]	RGB + IR	CFB	78.7	35.5	40.2
TCCDNet (ours)	RGB + IR	CSPDarknet53	**83.3**	**36.2**	**42.1**

Bold represents the best results.

**Table 3:** Comparison results for the LLVIP dataset.

Model	Modality	Backbone	mAP_50_	mAP_75_	mAP
YOLOv5 [42]	RGB	CSPDarknet53	90.8	51.9	50.5
YOLOv5 [42]	IR	CSPDarknet53	94.6	72.2	61.9
SSD [10]	RGB	VGG16	82.6	31.8	39.8
GAFF [43]	IR	VGG16	90.2	57.9	53.5
CCIFNet [34]	RGB + IR	ResNet50	**97.6**	72.6	63.6
CFT [44]	RGB + IR	CFB	97.5	72.9	63.6
TCCDNet (ours)	RGB + IR	CSPDarknet53	97.3	**78.4**	**67.8**

Bold represents the best results.

**Table 4:** Ablation results for the KAIST dataset.

Model	MR^−2^ (IoU = 0.5) (%)		
	All	Day	Night
Baseline	11.70	13.14	9.18
+CMC	10.28 (−1.42)	10.41 (−2.73)	10.17 (+0.99)
+DSFM	9.33 (−2.37)	10.33 (−2.81)	7.97 (−1.21)
+EMAC	8.89 (−2.81)	9.42 (−3.72)	8.17 (−1.01)
TCCDNet	7.87 (−3.83)	8.66 (−4.48)	6.43 (−2.75)

**Table 5:** Ablation results for the FLIR ADAS dataset.

Model	Precision	Recall	mAP_50_	mAP_75_	mAP
Baseline	82.4	67.7	79.7	31.3	38.9
+CMC	81.7 (−0.7)	70.4 (+3.3)	80.9 (+1.2)	34.2 (+2.9)	40.5 (+1.6)
+DSFM	84.8 (+2.4)	71.7 (+4.0)	83.1 (+3.4)	35.8 (+4.5)	41.8 (+2.9)
+EMAC	81.4 (−1.0)	75.8 (+7.1)	83.2 (+3.5)	37.2 (+5.9)	42.6 (+3.7)
TCCDNet	83.9 (+1.5)	72.5 (+4.8)	83.8 (+3.6)	36.2 (+4.9)	42.1 (+3.2)

**Table 6:** Ablation results for the LLVIP dataset.

Model	Precision	Recall	mAP_50_	mAP_75_	mAP
Baseline	93.9	89.3	95.4	73.2	63.7
+CMC	95.0 (+1.1)	92.7 (+3.4)	97.1 (+1.7)	79.1 (+5.9)	68.1 (+4.4)
+DSFM	94.5 (+0.6)	92.3 (+3.0)	96.9 (+1.5)	76.6 (+3.3)	66.2 (+2.4)
+EMAC	95.2 (+1.3)	92.4 (+3.1)	97.2 (+1.8)	77.5 (+4.3)	66.5 (+2.8)
TCCDNet	95.2 (+1.3)	93.1 (+3.8)	97.3 (+1.9)	78.4 (+5.2)	67.8 (+4.1)

## Data Availability

The datasets utilized in the experiments are publicly accessible as follows: the KAIST dataset is available at https://github.com/SoonminHwang/rgbt-ped-detection/tree/master/data (accessed on 10 September 2024), the LLVIP dataset can be accessed at https://bupt-ai-cz.github.io/LLVIP/ (accessed on 10 September 2024), and the FLIR ADAS dataset is available at https://www.flir.com/oem/adas/adas-dataset-form/ (accessed on 10 September 2024).
